# Cooperative Obstacle Avoidance for Multiple UAVs Using Spline_VO Method

**DOI:** 10.3390/s22051947

**Published:** 2022-03-02

**Authors:** Mingzhu Peng, Wei Meng

**Affiliations:** The Guangdong Province Key Laboratory of Intelligent Decision and Cooperative Control, School of Automation, Guangdong University of Technology, Guangzhou 510006, China; m15625094363@163.com

**Keywords:** multiple UAV, cooperative obstacle avoidance, velocity obstacle, cubic B-spline

## Abstract

In order to solve multiple unmanned aerial vehicle (UAV) dynamic collision avoidance, a cooperative obstacle avoidance algorithm considering UAV’s kinematic constraints has been developed. In the proposed algorithm, the useful information of UAVs is screened out by a Heartbeat information filtering mechanism and fused by the user datagram protocol (UDP) communication method, which improves communication performance among UAVs. In addition, the velocity obstacle (VO) method combined with cubic uniform B-spline curve is used to avoid obstacles and generate smooth paths, which can be applied to practical scenes. Finally, dynamic and static obstacle avoidance simulations are carried out to verify the effectiveness of the proposed algorithm.

## 1. Introduction

In the past decades, unmanned aerial vehicle(UAV) has been the subject of much discussion because of promising applications in military and civil fields, such as cooperative operations [1], electric power inspection [2], land mapping [3], and so on. A single UAV has limitations in terms of payload, endurance and mission capability, while a collaborative system composed of multi-UAV has obvious advantages in practicability and scalability [4]. One of the core problems of UAV group is navigation, i.e., how to reach the destination without colliding with dynamic and static obstacles. Therefore, cooperative obstacle avoidance of multi-UAV is an important direction of UAV development and application.

Many collision avoidance models for UAV have been proposed, which can be divided into two categories, i.e., model-free approaches and model-based approaches. The model-free approaches do not require exploring the model to recover the environments, which consist of sampling method, swarm intelligence, deep reinforcement learning, and so on. Early random road map and fast search tree methods are often used for sampling, but the planning process is very time consuming, and the methods are based on the assumption that the environment is static. These factors make it difficult to apply in practical engineering [5,6]. Swarm intelligence algorithms mainly include particle swarm optimization (PSO), ant colony optimization (ACO), artificial bee colony (ABC) algorithm and so on [7,8,9]. When these algorithms are used to deal with a large number of sample data, they will occupy more computer memory, and have a high demand on the running speed of the computer. As a research hotspot in machine learning, deep reinforcement learning can obtain effective control decisions for high-dimensional raw data input by relying on the perception ability of deep learning [10]. However, this system usually works in specific tasks and is difficult to be extended to other environments.

Model-based methods are designed based on accurate models, such as geometric model, artificial potential field method, velocity obstacle (VO) method and so on. However, due to the limitations of realistic environments and the constraints of UAV itself, many of the existing methods have a large amount of calculation and are not suitable for reactive collision avoidance of multi-vehicle systems [11]. Fortunately, VO method has the ability to predict the collision in advance, and the calculation requirements are not high, and it is more suitable for multi-obstacle avoidance in complex environments [12]. VO method was first proposed by Fiorini and Shiller in [13], which is a commonly used multi-robot local path planning method. The model needs to solve complex nonlinear optimization problems in the relative velocity space, it is difficult to guarantee the real-time performance, and does not solve the problem of safe and non-oscillation navigation among multiple agents in a large and chaotic environment [14]. Berg proposed the reciprocal velocity obstacles (RVO) based on VO, which solves the oscillation problem of the VO method [15]. However, the RVO has some limitations, in particular, it often causes agents to end up with a reciprocal dance  condition, as they cannot agree on which side to pass through the other [16]. A selective velocity obstacle (SVO) method is proposed in [17] for collision avoidance between multiple UAVs on the basis of VO method, and the right hand first principle is added in SVO for collision avoidance decision-making. Although the above approaches have had some success, there are still many limitations. For example, each UAV acts independently and makes decisions according to the local state information obtained by itself, which often fails to achieve the global optimal decision. The communication volume is too large during cooperative obstacle avoidance, resulting in redundancy and a lot of calculation. The kinematics constraints of UAVs in real environments are also not considered.

Based on the above factors, this paper proposes a feasible cooperative obstacle avoidance scheme for multi-UAV. The cooperative obstacle avoidance scheme uses user datagram protoco(UDP) and information completion algorithm to reduce communication traffic and share Heartbeat information of each UAV. Then, a Spline_VO method is used to realize cooperative obstacle avoidance for multiple UAVs and generate smooth flight path for each UAV. The scheme is simple in calculation, high in efficiency, in line with the kinematics law of the UAV, and can ensure that multi-UAV will reach the target smoothly.

The main contributions of our work are summarized as follows:

1. We present a feasible obstacle avoidance scheme, which can help UAV group to avoid dynamic and static obstacles smoothly and generate smooth flight path. The problems such as easy to fall into local optimization, communication redundancy and kinematics constraint of UAV are solved.
2. We propose a screening mechanism of UAV Heartbeat information, which can effectively avoid the interference of timeout signal and long-distance communication to UAV’s obstacle avoidance performance, improve the stability of obstacle avoidance and reduce communication volume.
3. For static obstacles, we also use Spline_VO method to avoid obstacles and smooth the path for polygonal obstacles. It is more in line with the situation that the UAV encounters static obstacles in the real environment.
4. We verified the effectiveness and performance of the proposed obstacle avoidance scheme by comparing it with other obstacle avoidance schemes in path and time.

The rest of this paper is organized as follows: Cooperative communication mechanism of UAV group is presented in Section 2. Section 3 introduces the obstacle avoidance of UAV based on the proposed communication algorithm. The simulation results are given in Section 4. Section 5 concludes this paper.

## 2. Cooperative Communication Mechanism for Multi-UAV

UAV communication is the key to cooperative obstacle avoidance. How to reduce communication time and ensure the accuracy of communication is one of the key points of our research. Transmission control protocol (TCP) requires three handshakes before data transmission, resulting in communication delay, while UDP has no delay in establishing connections and can reduce energy consumption through regular communication [18]. Therefore, in this study, each UAV communicates with others based on UDP broadcasting its Heartbeat [19] information. The main information contained in a UAV Heartbeat(HB) is shown in Table 1.

**Figure 1:**
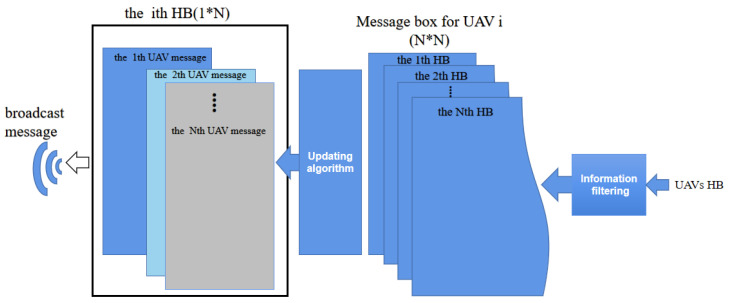
Information filtering and update. HB: Heartbeat.

In the UAV communication system, all UAVs regularly broadcast their HB information at a fixed frequency fHz, which will be stored in MessageBox. If there are *N* UAVs in the system, each UAV will receive N−1 HB messages from other UAVs regularly under normal communication conditions, while MessageBox will receive N×N HB messages every communication cycle, which will inevitably lead to information redundancy. In addition, due to the decline of long-distance communication or network quality, UAVs may not receive long-distance Heartbeat information, resulting in information loss, or the received signal times out and becomes useless signal, which greatly affects the obstacle avoidance performance of the UAV group. To solve these problems and ensure the real-time and accuracy of communication between UAVs, we proposed a HB information filtering mechanism (HIFM) to screen the Heartbeat signals broadcast by UAVs. The specific process of HIFM is shown in Figure 1.

HIFM firstly filters out useless information. When conducting collision avoidance, the UAV needs to know the horizontal and vertical distances between itself and other UAVs or obstacles. Set the minimum horizontal safety distance and the minimum vertical safety distance to avoid collision as $d_{min}$ and $h_{min}$, respectively. Once the minimum safety distance is reached, the UAV shall conduct collision avoidance operation. When the horizontal distance between two UAVs is greater than $d_{min}$ or the vertical distance is more than $h_{min}$, the HB of each other is regarded as useless information and will not be received. In addition, due to environmental interference or communication delay, a UAV at time *T* will refuse to receive the message broadcast before time T−3, which is also considered as useless information. After sifting through the information, the UAV *i* has the HB of k(k≤N) other UAVs in memory. The HB of *k* other UAVs is fused into a 1×N HB information matrix through the ID of UAVs, in which the unreceived HB of UAVs is empty.

In a network of *N* UAVs, the *i*th UAV will receive HB messages $C_{j}\left(j=1\cdots N,j\neq i\right)$ from other UAVs. In order to ensure the performance of obstacle avoidance, UAV *i* needs to check and update the HB from other UAVs in every communication cycle. If the HB $C_{i}$ of the *i*th UAV contains the HB of other *k* UAVs, check the HB $C_{1}$ of the first UAV and determine whether it contains the latest HB through GPS time, extract the latest HB and store it in the HB $C_{i}$ of the *i*th UAV. If the HB of the *k*th UAV contained in $C_{1}$ is empty or not the HB under the latest GPS time *T*, check the HB of the next UAV. Repeat the above process until the HB of other *k* UAVs contained in the HB of the *i*th UAV is updated. After several cycles, the latest HB needed for obstacle avoidance will be stored in the MessageBox of each UAV. The above steps are achieved through Algorithm 1, and $C_{i}^{j}$ represents the HB message of the *j*th UAV in the *i*th UAV.
**Algorithm 1** Information completion algorithm1:**Input**: HB of UAV $C_{i}$2:$\;\;\;\;\mathrm{HB of other UAV}C_{j}$, j=1,2,3.....N; j≠i3:**for**j≐1 to total UAVs **do**4:      **if** $C_{i}^{j}\neq null$ **then**5:             tem = 06:             **for** h≐1 to total UAVs **do**7:                   **if** GPS T of HB[h*total_UAV_Num8:                     +j]≥tmp **then**9:                          store HB[h*total_UAV_Num + j]10:                    information11:                        tem = GPS T of HB[h*total_UAV_Num12:                     + j]13:                 **end if**14:           **end for**15:      **end if**16:**end for**

We can represent the information update of the *i*th UAV by:

(1) $$M_{ki}\left(t+1\right)=\sum_{j}^{N}\rho_{ji}^{\left(k\right)}M_{ji}\left(t\right),k=1,2...,1\leq j\leq N,$$

where $M_{ji}$ represents the HB of the *j*th UAV received by the *i*th UAV. $\rho_{ji}^{\left(k\right)}$ is the weighting of the information fusion, indicating whether there is the latest information of the *k*th UAV in the HB of the *j*th UAV received in the *i*th UAV, given as:

(2) $$\rho_{ji}^{\left(k\right)}=\left\{\begin{array}{c} 1\;\mathrm{If}\;\mathrm{the}\;\mathrm{update}\;\mathrm{is}\;\mathrm{successful},\\ 0\;\mathrm{otherwise}. \end{array}\right.$$

All possible HB received by UAV $C_{i}$ from other UAVs can be obtained by Equations (1) and (2), which is defined as $M_{i}$,

(3) $$M_{i}\left(t+1\right)=A_{i}M_{i}\left(t\right),$$

where $M_{i}\left(t\right)={\left[M_{1i}\left(t\right),M_{2i}\left(t\right),\cdots ,M_{Ni}\left(t\right)\right]}^{T}$, and

(4) $$A_{i}=\left[\begin{array}{cccc} \rho_{1i}^{\left(1\right)} & \rho_{2i}^{\left(1\right)} & \cdots & \rho_{Ni}^{\left(1\right)}\\ \rho_{1i}^{\left(2\right)} & \rho_{2i}^{\left(2\right)} & \cdots & \rho_{Ni}^{\left(2\right)}\\ \vdots & \vdots & \vdots & \vdots \\ \rho_{1i}^{\left(N\right)} & \rho_{2i}^{\left(N\right)} & \cdots & \rho_{Ni}^{\left(N\right)} \end{array}\right]$$

It should be noted that since the UAV only updates and stores the effective obstacle avoidance information when other UAV $C_{j}$ is in the obstacle avoidance range, because of the update result, only one number in the weighting set {$\rho_{1i}^{k},\rho_{2i}^{k},\cdots ,\rho_{Ni}^{k}$} is equal to 1, and others are equal to 0. Otherwise, all elements of the weighting are set 0. The weighting set function $\sum_{j}^{N}\rho_{ji}^{\left(k\right)}$ denoted by:

(5) $$\sum_{j}^{N}\rho_{ji}^{\left(k\right)}=\left\{\begin{array}{c} 1\;\mathrm{If}\;\mathrm{within}\;\mathrm{obstacle}\;\mathrm{avoidance}\;\mathrm{range},\\ 0\;\mathrm{otherwise}. \end{array}\right.$$

Through the following proof, we can conclude that the information obtained by each UAV through multiple communications is convergent. In UAV communication Equations (3) and (4), when time *t* tends to *∞*:

(6) $$\begin{array}{cc} ∥M_{i}{\left(t+1\right)∥}_{t\to \infty } & =∥A_{i}M_{i}{\left(t\right)∥}_{t\to \infty }\\ \; & \leq ∥A_{i}{∥}_{\infty }{∥M_{i}\left(t\right)∥}_{\infty }\\ \; & \leq \sum_{j=1}^{N}\rho_{ji}^{\left(k\right)}{∥M_{i}\left(t\right)∥}_{\infty }\\ \; & \leq ∥M_{i}{\left(t\right)∥}_{\infty } \end{array}$$

It can be seen that information completion algorithm can be used to reduce information redundancy, and the weakly connected communication network can be processed through the protocol to realize the cooperation of multiple UAV systems.

## 3. Uav Obstacle Avoidance

### 3.1. Velocity Obstacle Method

VO is a common method for robots to avoid moving obstacles, and the detailed principle of VO method is described in [13], which has the following characteristics:

1. It is a geometric constraint representation method to avoid obstacles;
2. It can be used to deal with collision avoidance problems of multiple dynamic obstacles;
3. Dynamic constraints and maneuvering constraints are considered.

**Figure 2:**
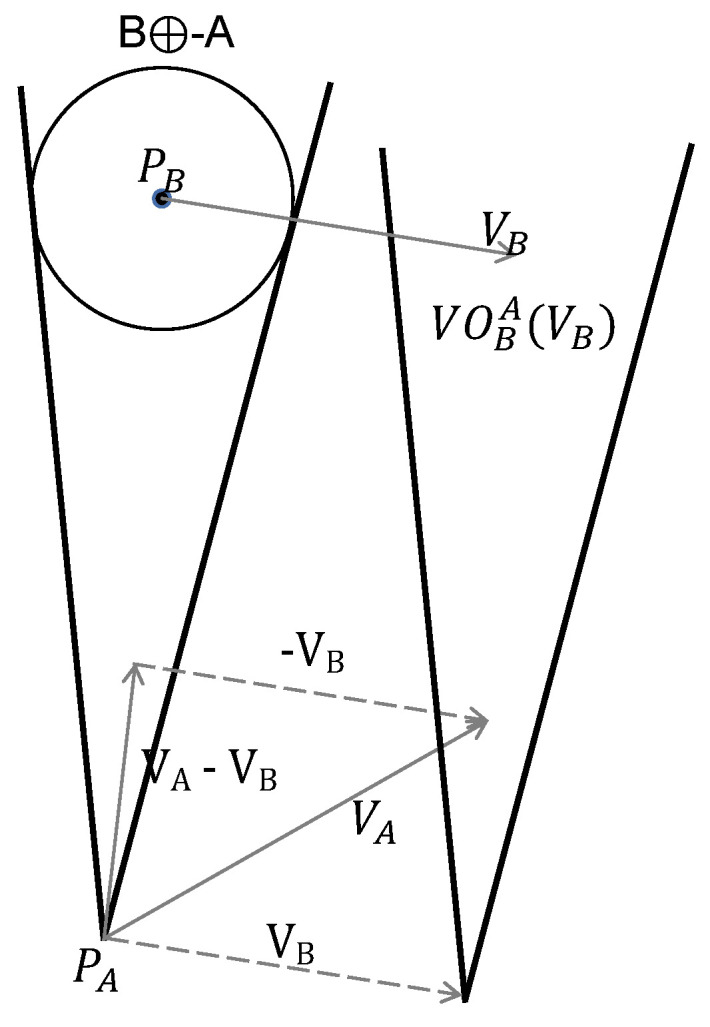
Geometric definition of VO.

VO can determine the track segment where there is an urgent threat from the distribution of surrounding obstacles perceived by the sensor. At the same time, VO can construct a VO cone according to the obtained speed and position information of the UAV and the intruder, and obtain the feasible velocity set of the UAV. The VO model is shown in Figure 2.

In Figure 2, A and B are two UAVs, their current position vectors are $P_{A}$ and $P_{B}$, respectively, and their speed vectors are $V_{A}$ and $V_{B}$, respectively. The VO cone of B to A can be defined by

(7) $$VO_{B}^{A}\left(V_{B}\right)=\left\{v_{A}|\lambda \left(P_{A},V_{A}-V_{B}\right)\cap \left(B⊕-A\right)\neq 0\right\}$$

The VO cone can be geometrically described as a cone with a vertex at $P_{A}$, $\lambda (P_{A},V_{A}-V_{B})$ and represents a ray from $P_{A}$ in the direction of $V_{A}-V_{B}$. If $V_{A}\subseteq VO_{B}^{A}\left(V_{B}\right)$, A and B will collide at some point in the future. Therefore, if the speed of the UAV is within the area of the VO cone, the speed vector outside the area should be selected, so that the UAV can avoid the obstacle and continue moving.

### 3.2. Spline _VO Method to Avoid Obstacles

**Figure 3:**
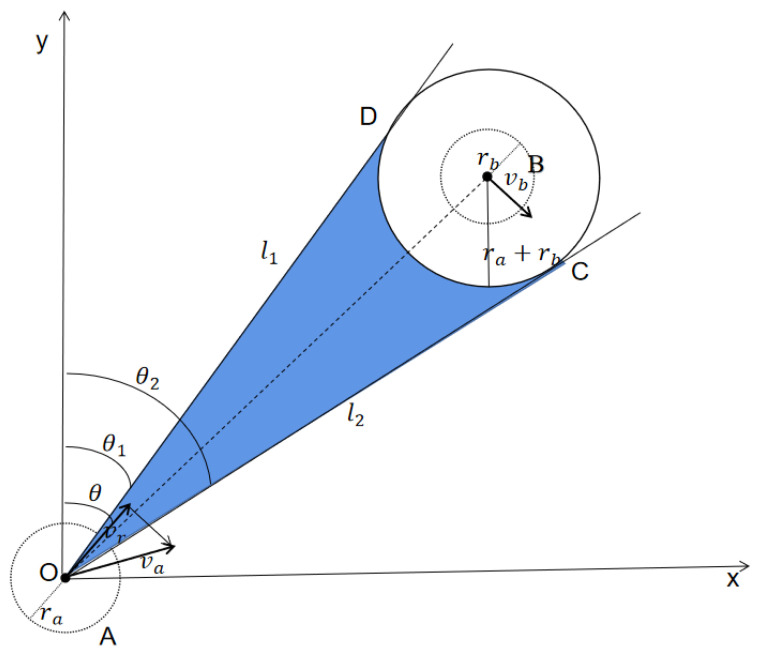
Collision avoidance model of UAV.

During the task execution, the UAV should detect the constantly changing environment around it in real time, perceive the speed and position of other UAVs and dynamic obstacles to make obstacle avoidance judgment, as shown in Figure 3. There are two UAVs A and B at $P_{A}$ and $P_{B}$ with radii $r_{a}$ and $r_{b}$, respectively. Generally, UAV A is simplified as a particle and UAV B is expanded into a circle with radius $r_{a}+r_{b}$ in VO method.

As shown in Figure 3, the velocity vector of UAV A is $v_{a}$, and that of UAV B is $v_{b}$. The speed of UAV B relative to UAV A is $v_{r}=v_{a}-v_{b}$. UAV B can be regarded as stationary, and UAV A moves relative to B at the speed of $v_{r}$. Assume that the velocity vectors of A and B do not change, and moving at a constant velocity. If the relative velocity is within the blue area, which is called the collision cone, then at some point in the future, UAV A and B will collide. Therefore, the core of the VO method is to determine the blue area.

Assume that the horizontal coordinate of UAV A at the current moment is ($X_{a}$, $Y_{a}$), and the coordinate of UAV B is ($X_{b}$, $Y_{b}$). Connect $P_{a}$ and $P_{b}$, and draw tangent lines $l_{1}$ and $l_{2}$ to UAV B from point $P_{a}$. $\theta_{1}$ is given by Equation (8).

(8) $$\theta_{1}=\theta_{\mathrm{OB}}-∠\mathrm{BOD}$$

where $\theta_{\mathrm{OB}}$ is the angle between the line OB and the *Y*-axis, and ∠BOD can get from the UAVs coordinates:

(9) $$\theta_{\mathrm{OB}}=\arctan\frac{X_{a}-X_{b}}{Y_{a}-Y_{b}}$$

(10) $$∠\mathrm{BOD}=\arcsin\frac{\left(r_{a}+r_{b}\right)}{\sqrt{{\left(Y_{a}-Y_{b}\right)}^{2}+{\left(X_{a}-X_{b}\right)}^{2}}}$$

The obstacle avoidance angle $\theta_{1}$ and $\theta_{2}$ can be obtained through the above equations.

(11) $$\theta_{1}=\arctan\frac{X_{a}-X_{b}}{Y_{a}-Y_{b}}-\arcsin\frac{\left(r_{a}+r_{b}\right)}{\sqrt{{\left(Y_{a}-Y_{b}\right)}^{2}+{\left(X_{a}-X_{b}\right)}^{2}}}$$

(12) $$\theta_{2}=\arctan\frac{X_{a}-X_{b}}{Y_{a}-Y_{b}}+\arcsin\frac{\left(r_{a}+r_{b}\right)}{\sqrt{{\left(Y_{a}-Y_{b}\right)}^{2}+{\left(X_{a}-X_{b}\right)}^{2}}}$$

According to the VO method, the two UAVs A and B will collide if $\theta_{1}<\theta <\theta_{2}$. Therefore, in order to avoid collision, it should be true that $\theta ⊈[\theta_{1},\theta_{2}]$. At this point, the UAV selects the feasible speed and generates a new speed $V_{new}$ according to the lowest cost.

Considering the kinematic constraints of a UAV in the real world, the motion path of a UAV should be smooth. The B-spline curve is a powerful tool for path smoothing, which can generate the desired trajectory for different times and obstacle configurations. The basic principle of B-spline is as follows [20].

(13) $$\begin{array}{c} G_{i,n(t)}=\frac{1}{n!}\sum_{j=0}^{n-i}{\left(-1\right)}^{j}C_{n+1}^{j}{\left(t+n-1-j\right)}_{n}\\ \;\;t\in [0,1],i=1,2,\cdots ,n \end{array}$$

where *n* is the number of B-splines, so the cubic uniform B-spline can be written as:

(14) $$\left\{\begin{array}{c} G_{0,3}\left(t\right)=\frac{1}{6}\left(-t^{3}+3t^{2}-3t+1\right)\\ G_{1,3}\left(t\right)=\frac{1}{6}\left(3t^{3}-6t^{2}+4\right)\\ G_{2,3}\left(t\right)=\frac{1}{6}\left(-3t^{3}+3t^{2}+3t+1\right)\\ G_{3,3}\left(t\right)=\frac{1}{6}t^{3} \end{array}\right.$$

Thus, the cubic uniform B-spline segment $P_{0,3}\left(t\right)$ can be described as

(15) $$P_{0,3}\left(t\right)=\frac{1}{6}{\left[\begin{array}{c} 1\\ t\\ t^{2}\\ t^{3} \end{array}\right]}^{T}\left[\begin{array}{cccc} 1 & 4 & 1 & 0\\ -3 & 0 & 3 & 0\\ 3 & -6 & 3 & 0\\ -1 & 3 & -3 & 1 \end{array}\right]\left[\begin{array}{c} p_{0}\\ p_{1}\\ p_{2}\\ p_{3} \end{array}\right],t\in \left[0,1\right]$$

where $p_{0}$, $p_{1}$, $p_{2}$ and $p_{3}$ are historical locus points, current locus points, and next locus points, respectively.

In this paper, cubic B-spline and VO method are used to smooth the path while avoiding obstacles. For UAV, since the historical waypoints are known, and the information of obstacles in the local range can be obtained through the UAV sensor and communication, $V_{new}$ is selected according to VO method first. Hence, one or more waypoints that the UAV is about to pass through can be estimated in real time through the UAV’s current position and $V_{new}$. Next, we use the historical trajectory points of the UAV, the trajectory points at the current moment, and the trajectory points at the next moment after the speed changes to generate a set of b-spline curves. Then, a series of curve points satisfying the cubic B-spline can be obtained by Equation (15), and the path smoothness can be realized when the UAV avoids obstacles.

### 3.3. Irregular Static Obstacle Collision Avoidance

When encountering a circular obstacle, set $V_{b}$ equal to 0. However, when encountering irregular static obstacles, it will cost more to uniformly set obstacles as circular ones. Therefore, the VO method is improved to avoid polygonal obstacles at a lower cost [21]. Static polygons can generally be divided into two types: convex and non-convex, while a non-convex polygon can be considered to consist of several convex polygons. Therefore, the key to avoid irregular static obstacles is how to avoid convex polygon obstacles.

When the sensor carried by the UAV detects an obstacle, the detected part will be constructed with a convex polygon. When the *m* vertices ($x_{0}$, $y_{0}$), ($x_{1}$, $y_{1}$)…, ($x_{m-1}$, $y_{m-1}$) of the obstacle are detected, a convex polygon with the centroid denoted as ($C_{x}$, $C_{y}$) is constructed. Their values are calculated by the following formula:

(16) $$\begin{array}{cc} \; & C_{x}=\frac{1}{6S}\sum_{i=0}^{m-1}\left(x_{i}+x_{i+1}\right)\left(x_{i}y_{i+1}-x_{i+1}y_{i}\right),\\ \; & C_{y}=\frac{1}{6S}\sum_{i=0}^{m-1}\left(y_{i}+y_{i+1}\right)\left(x_{i}y_{i+1}-x_{i+1}y_{i}\right) \end{array}$$

where *S* represents the area of the constructed convex polygon, which can be obtained from Equation (17).

(17) $$S=\frac{1}{2}\sum_{i=0}^{m-1}\left(x_{i}y_{i+1}-x_{i+1}y_{i}\right)$$

**Figure 4:**
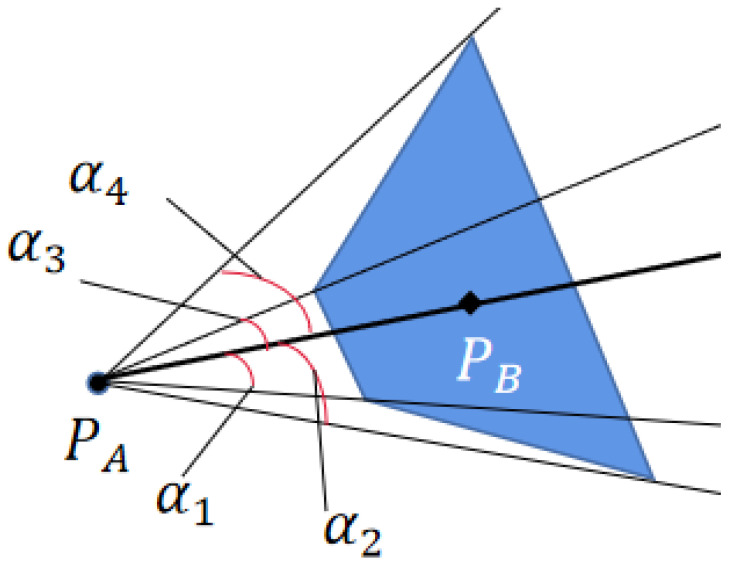
Convex polygon collision avoidance model.

As shown in Figure 4, two half spaces can be formed by connecting the position $P_{A}$ of the UAV with the centroid $P_{B}$ of the polygon. From the angle collected in each half space, we can obtain a maximum angle, thus forming the collision cone of polygon. If the UAV’s speed is selected from outside the collision cone, the obstacles will be avoided.

### 3.4. Cooperative Obstacle Avoidance Algorithm for Multiple UAVs

**Figure 5:**
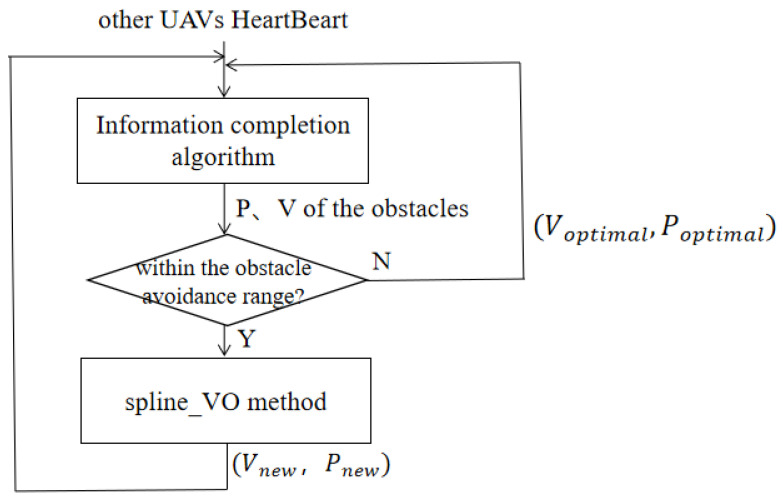
Multi UAVs cooperative obstacle avoidance process.

In this paper, communication between multiple UAVs is firstly achieved through information completion algorithm, and then smooth obstacle avoidance is achieved by Spline_VO method. Specific steps to realize cooperative obstacle avoidance algorithm of multiple UAVs are shown in Figure 5.

In this paper, $V_{optimal}$ is determined by the vector of the current pathpoint and the target point, and is constrained by the maximum velocity and acceleration. When the new speed $V_{new}$ is selected using the Spline_VO method, the speed with the least deviation from the $V_{optimal}$ is selected based on the feasible speed set. Then, the $V_{new}$ is sent to the UAV’s flight control board to update its position.

## 4. Simulation Results

In order to verify the effectiveness of our proposed cooperative obstacle avoidance algorithm, we use the communication and obstacle avoidance methods described in this paper to give the simulation results of cooperative obstacle avoidance flight of multiple UAVs, and give the comparison of the simulation results of UAV flight path before and after using B-spline to do path smoothing.

The experimental environment has a circular obstacle and a rectangle (convex polygon). In order to fully test the UAV’s obstacle avoidance performance, a simulation algorithm space with size 300 m × 300 m is set, where the algorithm parameters are $v_{max}$ = 5 m/s, $d_{saft}$ = 10 m, $d_{com}^{max}$ = 80 m and $h_{vertical}$ = 30 m. $v_{max}$ represents the maximum flight speed of a UAV, $d_{saft}$ is the safe flight distance between a UAV and obstacles, $d_{com}^{max}$ is the maximum communication range of a UAV, and $h_{vertical}$ represents the flight altitude of all UAVs. The starting and ending points of the UAV group in the horizontal axis are presented in Table 2.

In the case of the same simulation parameters, the information completion algorithm proposed in this paper is used to compare the communication of multiple UAVs with the direct UDP communication and search algorithm. Three path diagrams were generated showing the situation of five UAVs using different communication methods to avoid each other and reach the target point. In addition, after 10 simulation tests, the total time of 5 UAVs arriving at the target point is counted, and the total time of arriving at the target point is the average of 10 experiments.

**Figure 6:**
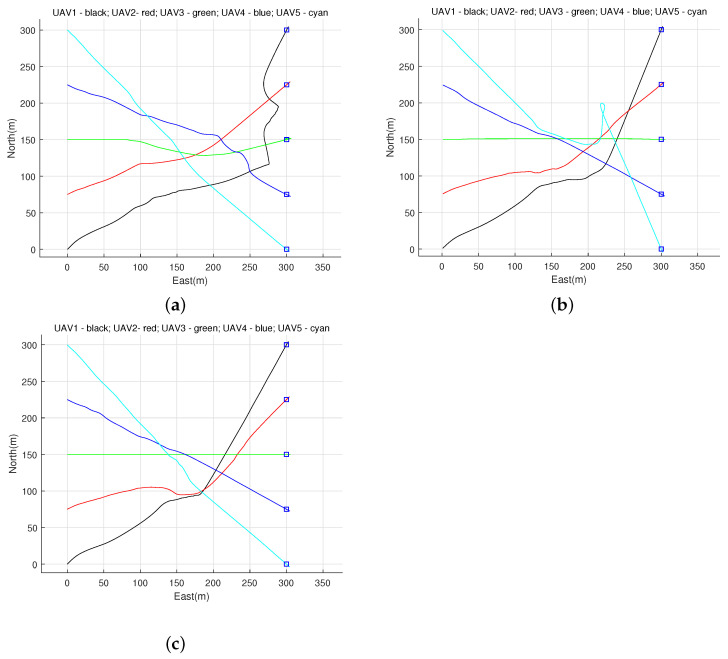
Path comparison using different communication modes. (**a**) Use UDP for direct communication. (**b**) Use search algorithm to communicate [19]. (**c**) Use Information completion algorithm to communicate.

According to the comparison of paths generated by different communication methods in Figure 6 and the comparison of data in Table 3, it can be seen that the information completion algorithm filters useless heartbeat information and stores the latest heartbeat information more effectively, which improves the communication speed and accuracy of UAVs, thus optimizing the obstacle avoidance path of UAVs and speeding up the arrival time of UAVs.

**Figure 7:**
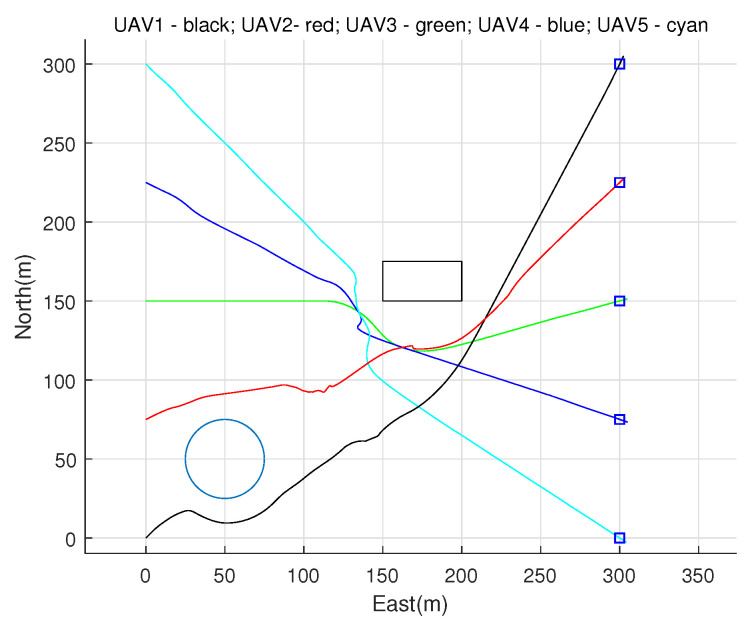
Simulation results of cooperative obstacle avoidance based on proposed.

**Figure 8:**
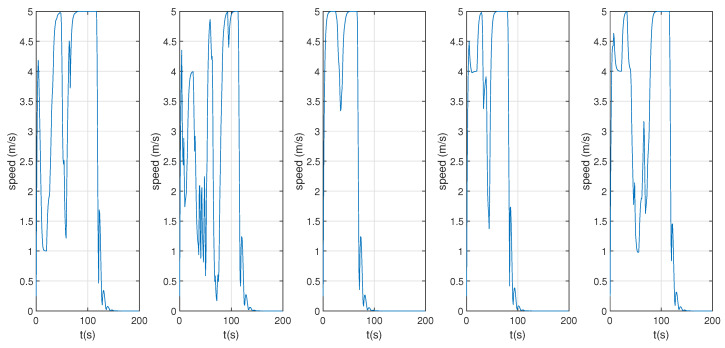
Obstacle avoidance speed of 5 UAVs.

**Figure 9:**
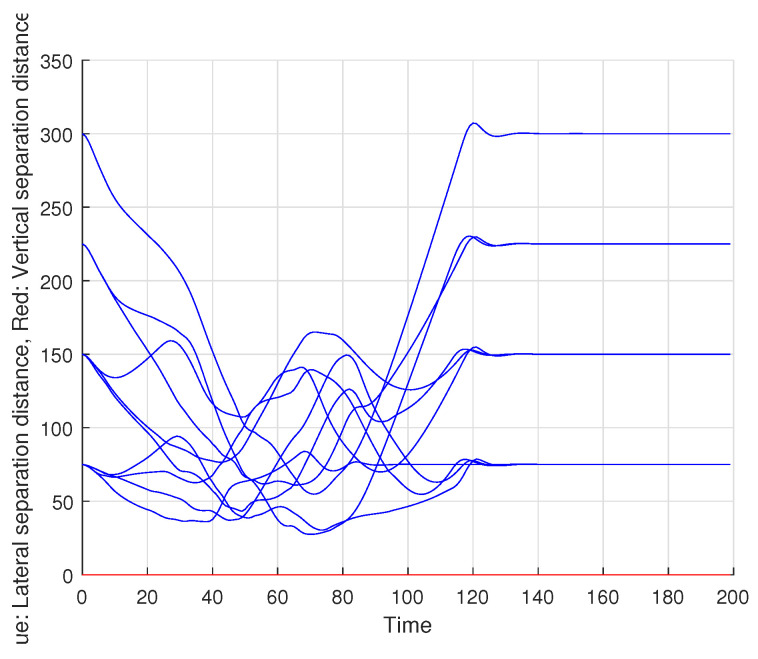
Horizontal distance profile of UAVs.

Figure 7 shows the result of each UAV’s obstacle avoidance performance through cooperative communication and Spline_VO method, and the obstacle avoidance path of multiple UAVs is generated. Figure 8 shows the flying speed of five UAVs. Figure 9 is the horizontal distance profile between UAV *i* and UAV *j*.

As can be seen from Figure 7, Figure 8 and Figure 9, a collision-free path of a multiple UAVs can be generated by using the proposed cooperative obstacle avoidance algorithm, and the distance between UAVs is greater than the safe flight distance. Therefore, we can claim that the cooperative obstacle avoidance method we put forward is effective.

**Figure 10:**
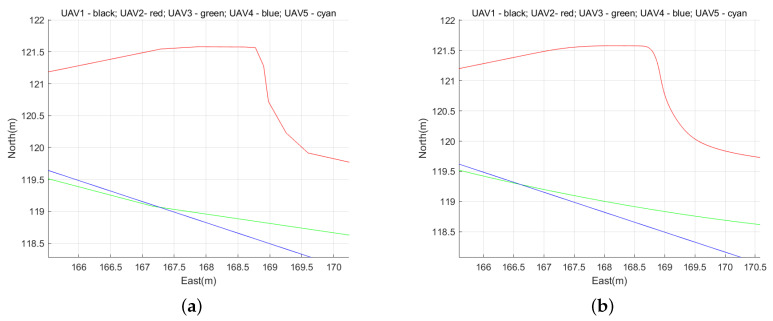
Loca amplification of path. (**a**) Local amplification of path before smoothing. (**b**) Local amplification of path after smoothing.

However, the trajectory of single UAV in obstacle avoidance has problems of too large turning angle and uneven path, so the spline_VO method is introduced to solve these problems. To show the effect of the spline_VO method smoothing the path, we zoomed in on the flight paths of two UAVs, and plotted Figure 10. Figure 10a shows the simulation part of UAV’s path before smoothing, and Figure 10b shows the simulation part of UAV’s path after smoothing.

According to the comparison between Figure 10a,b, the Spline_VO method does solve the problems that UAV’s turning angle is too large and the path of UAV is not smooth.

## 5. Conclusions

In this paper, a cooperative obstacle avoidance algorithm of UAV group is proposed. Based on this algorithm, multiple UAVs can avoid dynamic obstacles and irregular static obstacles during the task execution from the start point to the end point. Heartbeat information filtering mechanism is proposed to solve the communication problems of multiple UAVs. Furthermore, the spline_VO method is used to achieve the obstacle avoidance and path smoothing of multiple UAVs, which allows UAVs to be deployed in real-world work environments. The simulation experiments is completed, and the performance of the proposed cooperative obstacle avoidance algorithm in cooperative obstacle avoidance and the feasibility of application in real environments are proved.

## Figures and Tables

**Table 1:** Unmanned aerial vehicle (UAV) $\left(C_{i}\right)$ Heartbeat.

UAV ID
UAV adjacent number (UAV valency)
Total UAV number in network
x position
y position
z position
Vx speed
Vy speed
Vz speed
GPS time
UAV mode
Replacement UAV
Target ID
Want to target ID
Cost
UAV status

**Table 2:** UAVs starting and target points.

UAV ID	Initial Path Point	Target Waypoints
1	(0, 0)	(300 m, 300 m)
2	(0, 75 m)	(300 m, 225 m)
3	(0, 150 m)	(300 m, 150 m)
4	(0, 225 m)	(300 m, 75 m)
5	(0, 300 m)	(300 m, 0)

**Table 3:** Total time comparison of UAVs to target point.

Communication Mode	Total Obstacle Avoidance Time (s)
Direct communication	669
Search algorithm	616
Information Completion algorithm	585

## Data Availability

Not applicable.
